# Prevalence and associated factors of anemia among adolescent girls attending high schools in Dembia District, Northwest Ethiopia, 2017

**DOI:** 10.1186/s13690-018-0324-y

**Published:** 2018-12-21

**Authors:** Kedir Abdela Gonete, Amare Tariku, Sintayehu Daba Wami, Terefe Derso

**Affiliations:** 1 Department of Human Nutrition, Institute of Public Health, College of Medicine and Health Sciences, University of Gondar, Gondar, Ethiopia; 2 Department of Environmental and Occupational Health and Safety, Institute of Public Health, College of Medicine and Health Sciences, University of Gondar, Gondar, Ethiopia

**Keywords:** Anemia, Adolescent girls, Ethiopia

## Abstract

**Introduction:**

Anemia is a global public health problem affecting both developing and developed countries. In Ethiopia, Adolescent girls are more vulnerable to anemia because of gender norms can leave girls disproportionately impacted by food insecurity, increased iron requirements related to their rapid growth, and menstrual loss. However, evidence on the problem is scarce because it has not been given due attention in Ethiopia. Therefore, this study assessed the prevalence and associated factors of anemia among late adolescent girls attending high schools in Dembia District, northwest Ethiopia.

**Methods:**

A school based cross-sectional study was conducted in Dembia District from March 1 to April 30/ 2017. Out of the randomly selected three high schools, 462 adolescents were included using the simple random sampling technique. A Standardized structured questionnaire was used to collect data. Capillary blood samples were drawn from adolescents using a portable Hb201+ instrument to measure hemoglobin. A bivariate and multivariable binary logistic regression analyses were employed to identify factors associated with anemia. Adjusted Odds Ratio (AOR) with a corresponding 95% Confidence Interval (CI) was computed to show the strength of associations.

**Results:**

The overall prevalence of anaemia among adolescent girls was 25.5%, (95%CI, 21.4, and 29.2). Of the total anemic adolescents, 109(92.4%) had mild anaemia, while 7(5.9%) and 2(1.7%) were found with moderate and severe anaemia, respectively. Dietary diversity score ((AOR =4.2(95% CI;1.7, 10.5)), household food security status ((AOR = 4.1(95% CI; 1.3, 13.2)), living status of adolescents with either of the two parents((AOR = 2;(95%CI;1.14,3.6)) and guardians ((AOR = 2.4;(95% CI;1.02,5.6)) showed statistically significant association with anemia.

**Conclusion:**

Anemia is a moderate public health problem in Dembia District. Dietary diversity score, household food security status, and living status of adolescents were the key determinants of anemia. Therefore, the government should focus on preventing food insecurity with increasing productivity to improve dietary diversification of the adolescent girls.

**Trial registration:**

Retrospectively registered.

## Background

Recently, adolescence has been considered as a critical window and a gateway to address the intergenerational cycle of malnutrition as adolescent girls enter pregnancy with poor nutritional reserve and give birth to undernourished babies [Lancet 2023]. Nearly 1.2 billion of the global population is comprised of adolescents 90% of which live in low or middle-income countries [1, 2]. Anemia is a nutritional disorder resulting when the number and size of red blood cells or the hemoglobin concentration falls below the established cut-off value, which consequently impairs the capacity of the blood to transport oxygen to the body [3–5]. It is a global public health problem affecting both developing and developed countries with its varied adverse consequences on health as well as on the socio- economic development of the countries [4, 6–8]. The most common cause of anemia worldwide is iron deficiency, resulting from prolonged negative iron balance, caused by inadequate dietary iron intake or absorption, increased needs for iron during pregnancy or growth periods, and increased iron losses as a result of menstruation and helminth (intestinal worms) infestation. Other important causes of anemia worldwide include infections, other nutritional deficiencies (especially folate and vitamins B12, A and C) and genetic conditions (including sickle cell disease, thalassemia – an inherited blood disorder – and chronic inflammation) and severe malaria and may be associated with secondary bacterial infection [4, 9, 10]. Adolescent girls are the vulnerable group to anemia because of increased iron requirements to support their rapid growth and mental development and replenish loss due to menstruation [11, 12] .

Experimental studies show that iron deficiency (ID) is capable of causing cognitive impairment in animals and humans, with brain mitochondrial damage as a basis for these alterations (). Among the cognitive impairments caused by iron deficiency (ID), those related to attention span, intelligence, and sensory perception functions are mainly cited, as well as those related to emotions and behavior. Generally, these impairments have been related to iron deficiency anemia (IDA) [13, 14].

The World Health Organization (WHO) estimated that more than two billion people about a quarter of the world’s are affected by anemia [4]. About 29.4% of women in the reproductive age have anemia [15]. The majority of south Asian adolescent girls were anemic; for instance, anemia was detected among 70, 51.8 and 67.7%of adolescents in Bangladesh, India, and Nepal, respectively [16]. In the further, adolescence anemia will be contribute to high maternal mortality, increased incidence of low birth weight, perinatal mortality,and fetal loss [17].

According to the 2011 Ethiopian Demographic Health Survey (EDHS) report, 13.4% of the adolescent girls were found with anemia [18]. Similarly, different district level studies showed a high prevalence (15.2–32%) of adolescent anemia [19–21].

Although nutritional anemia affects both sexes and all age groups, the problem is more prevalent among adolescent girls [22]. Furthermore, malaria, intestinal parasitic infections, Tuberculosis, and pneumonia [23] are some of the morbidity related determinates of anemia. On the other hand, poor economic status [24], the type of family [12, 25],residence [19, 20], family size, age [19, 21], large number of children [26] and occupation are the socio-demographic factors associated to anemia [21].

In 2012, the World Health Assembly endorsed a 50% reduction in the burden of anemia in women of reproductive age [5]. Ethiopia has also been striving to curve the high burden of micronutrient deficiencies, including anemia, through implementing national programs and strategies, such as the National Nutrition Program (NNP) and the micronutrient deficiency control strategy. However, these efforts targeted only pregnant mothers and children (6–59 months) through providing universal prenatal iron foliate supplementation and de-worming drugs [18, 27]. But more recently, the revised NNP has considered adolescent nutrition as one of the critical focus. Therefore, regular investigations of anemia among adolescents helps to make evidence based decisions. Nevertheless, there has been a scarcity of literature in Ethiopia, including the study area, Dembia District. Thus, this study aimed to assess the prevalence of anemia and associated factors among adolescent school girls in dembia District, northwest Ethiopia.

## Methods

### Study design and settings

A school based cross-sectional study was conducted from March 1 to April 15 /2017 among late adolescent girls aged 15 to 19 years. Dembia, the study area, is 765 km from the capital city of Ethiopia, Addis Abeba. The district has 45 kebeles (*smallest administrative unit in Ethiopia*) 7 high and 135 elementary schools. A total of 5071 adolescent girls were found attending high schools in the district. One hospital, 10 health centers, and 40 health posts were responsible for the health care.

### Study participants and sampling procedure

Adolescent girls attending high schools were included in the study. The sample size was calculated using the single population proportion formula by taking the level of confidence at 95%, margin of error 4%, design effect 1.5, non-response rate 10%,and expected prevalence 13.4% from EDHS [18]. Finally, a sample size of 462was obtained. Regarding the sampling procedures, a multi-stage sampling technique was employed and out of the total 7 high schools, three were selected using the lottery method. According to the school 2304 girls were attending the three high schools at the moment and proportional allocation was used to determine the number of students from each high school. School rosters were used as sampling frames and 181, 241 and, 40 adolescent girls from Kolladba, Chuahit and Sankisa kebele High schools were selected, respectively by using a systematic sampling technique. Adolescents who were pregnant and on treatment for anemia were excluded.

### Data collection procedure and management

A structured questionnaire, laboratory investigation for hemoglobin, anthropometric measurement for body mass index (BMI), and a standardized food security questionnaire from FANTA 2007 were used to collect data. Socio-demographic factors (age, marital status, occupation, religion, occupations, educational status of father and mother, birth order, birth interval, ethnicity and wealth index);health condition (malaria, intestinal parasite, menorragia),and dietary diversity (using 24 h recall methods) in the past 24 h in the schools were addressed for adolescents.

Families were interviewed about the socio-demographic and economic characteristics of mothers or guardians, while house-hold food security, wealth index, and environmental sanitation and hygienic practice were gathered through face to face interviews. The questionnaire was prepared in English and translated into Amharic and retranslated to English by language experts to ensure consistency. It was pretested on 5% of the sample out of the actual study setting. Three laboratory technicians, nine clinical nurses, and three heath officer supervisors participated as data collectors. Two daysˈ training was given to data collectors and supervisors on the objectives and methodology of the study and the process of data collection by the principal investigator. Throughout the course of data collection, collectors were supervised at each site and regular meetings of data collectors, supervisor and the principal investigator were held. The daily collected data was checked for accuracy. Data cleaning and crosschecking were made by the principal investigator and 10% was double entered to control errors during the entry.

### Blood collection and anthropometric measurement

Adolescent hemoglobin status was measured by using a portable battery-operated photometer (Hemacue M + 201). Capillary blood sample was taken by pricking the tip of the adolescent finger in a aseptic way. After rubbing the fingertip with sterile cotton, (immersed in alcohol) a 10 micro liter blood sample was collected by finger pricking with a sterile disposable lancet and the second blood drop was taken for hemoglobin measurement. Result was read within one minute. The photometer was calibrated before every session using provided standard. Hemoglobin level determination was done by trained laboratory technicians working out of the district.

Anthropometric measurement (height and weight) was taken according to World Health Organization (WHO) standard. Height was measured using a stadiometer and recorded to the nearest 0.1 cm. During the measurement, prominent body parts of the girls (occipital, shoulder, buttocks and heel) touched the stadiometer; shoes were taken off and they stood in Frankfurt position. Weight was measured with the Seca beam balance and recorded to the nearest 0.1 kg. Heavy clothes and shoes were taken off.

### Ethical consideration

Ethical approval for the survey was obtained from the Ethical Review Board of the University of Gondar. Written informed consent and assent was obtained from the adolescents and mothers of the selected participants. In case of illiterate mothers, consent was documented by thumbprint on the consent form, while literate ones signed the forms. All names and personal information regarding participants were kept confidential, and the data set for analysis was kept unidentified, using code numbers.

### Description of variables

According to WHO,the adolescent girl’s anemia status was considered as the outcome variable and was defined as individual hemoglobin levels below 12 g/dl at sea level and 11–11.9 mg/dl,8–10.9 g/dl, and lower than 8 g/dl were considered as mild, moderate and severe anemia respectively [28].Independent variables considered in this study were identified by reviewing previous literature (Table 1).

**Table 1:** Description of independent variable

Variables	Description
1. House hold level factor	
Area of residency	Urban and rural
socio economic status of the house hold	SES was measured by using tertile index derived from household assets and utilities score,the wealth tertile divided into (rich, middle and poor)
house hold food insecurity	HFAS(house hold food security access scale) was assessed from FANTA(Food and nutrition technical assistance) 2007 with nine main question, HFAS divided into (Food security defines the Household food security level of the summations was ≤1 point out of 27 scores while, the household food security level of the summations ≥2 point out of 27 scores were food insecure.)
Environmental sanitation and hygiene	Were included using WASH(Water Sanitation and Hygiene) formats.
2. Adolescent level	
Dietary diversity score	DDS was assessed by using FANTA 2016 with the classification of,the individual who was consume five and above defined food groups out of ten was adequate dietary diversity and The individual who was consume below five defined food groups in the previous day or night were inadequate dietary diversity
Adolescent BMI for age	Was calculated by using height and weight measurement with anthro-plus software and found BAZ (body mass index for age in z score)(thinness)
Adolescent menstrual status	Menstrual status especially, mennorhagia was assessed with the amount of pad changed within the hour and the individuals who were changed two and more pad within a hour .
Adolescent hemoglobin status	hemoglobin levels below 12 g/dl was anemic at sea level
3. Adolescent mother/Guardians	
Maternal education	Years of education completed
Maternal occupation	Years of education completed

### Statistical analysis

All the filled questionnaire and laboratory results were checked manually for completeness and consistency of responses. The collected data were coded and entered into EPI INFO version 7 and exported to SPSS version 20.0 for further analysis. The anthroplus nutritional software was used to determine the BMI of the adolescents with age, and the principal component analysis (PCA) was used for wealth index analysis. Adjusted Hb(hemoglobin) concentration was calculated as Hb = − 0.32× (altitude in meters × 0.0033) + 0.22× (altitude in meters × 0.0033)^2^ to subtract the adjustment from the measured Hb concentration at the relevant altitude (2200 m above the sea level) to get the sea-level value.

Descriptive statistics of the demographic characteristics of respondents and other factors were computed. Tables, graphs, means and frequencies were used to present information.

The binary logistic model was fitted to identify factors associated with anemia. Bivariate logistic regression analysis was performed, and variables with *p*-values < 0.2 were exported to the multivariable logistic regression analysis. Significance level was obtained at p-value of < 0.05.The Adjusted odds ratio was used for measuring the strength of the association. Crombachalpha (0.79) was calculated to check the internal consistency or reliability of the tool before data entry. The hosmer and lemshow test was done, and the result was 0.94, suggesting that the model fits the data well.

## Result

A total of 462 adolescent girls and their mothers participated in the study with a response rate of 100%. The mean age with a standard deviation of the adolescents was 17 ± 1.2. Half, 262(56.7%) of the adolescents were aged 17 to 19 years. More than half of the mothers and the adolescent girls 264(57.1%) lived in rural areas, and surprisingly 7.4% of the adolescents were married at the moment. Nearly two-thirds 272 (58.9%) of the mothers were not able to read and write, whereas almost half of the fathers 223 (48.3%) were able to read and write. About 42.9% the households were the in poor tertile (Table 2).

**Table 2:** Socio-Demographic and economic characteristics of the adolescents girls and their mother, Dembia District, Northwest Ethiopia, 2017

Characteristics	Frequency	Percent
Age of adolescent		
15–16	200	43.3
17–19	262	56.7
Age of mother		
25–34	74	16
35–44	257	55.6
45–54	110	23.8
≥ 55	21	4.5
Residency of adolescents		
Urban	198	42.9
Rural	264	57.1
Living residents of the Adolescents		
With family	382	82.7
With rent	80	17.3
Living status of the Adolescents		
With both mother and father	369	79.9
With only Mother	56	12.1
With only Father	12	2.6
With Guardians	18	3.9
Others	7	1.5
Mother education		
Not able to read and write	272	58.9
Able to read and write	151	32.7
Primary school	7	1.5
High school	7	1.5
Certificate and above	25	5.4
Father education		
Not able to read and write	181	39.2
Able to read and write	223	48.3
Primary school	3	0.6
High school	2	0.4
Certificate and above	35	7.6
Birth order		
1–5	393	85.1
6–10	69	14.9
Birth interval		
00–6	449	97.2
7–12	13	2.8
Marital status of the adolescents		
Single	425	92
Married	34	7.4
Divorced	3	0.6
House hold wealth Index		
Poor	198	42.9
Middle	151	32.7
Riche	113	24.5

According to the self-reported health status of the adolescents, 165 (35.7%) and 33 (7.1%) had upper respiratory tract and diarrheal morbidities, respectively. Cardiac diseases was reported by 16 (3.5%) of the participants, and 49 (10.6%) adolescents had malaria in the past two weeks. Also, 419 (90.7%) and 38(8.2%) of the girls started menstruation and were on menstruation during the data collection period, respectively. Three hundred seventy-seven (81.6%) of the adolescents had no massive bleeding disorder during menstruation cycles (Table 3).

**Table 3:** The Health related characteristics of the Adolescents in Dembia District, Northwest Ethiopia 2017

Characteristics	Frequency(n)	Percent (%)
Known chronic Diseases		
no	433	93.7
yes	29	6.3
Types of chronic Diseases		
Tuberculosis	6	1.3
Hypertension	1	0.2
Cardiac Diseases	16	3.5
kidney	6	1.3
Diarrheal episode		
no	429	92.9
yes	33	7.1
Upper respiratory tract		
no	297	64.3
yes	165	35.7
Infectious Diseases		
Malaria	49	10.6
Beginning of menstruation		
no	43	9.3
yes	419	90.7
Current status of menstruation		
no	380	82.3
yes	38	8.2
Changing of pad in a hour		
one times	22	4.8
two times	20	4.3
three times	1	0.2
nothing	377	81.6

Nearly three-fourths 304 (65.8%) of the adolescents had a meal frequency of three times per day. During the data collection, nearly half 211 (45.7%) of the adolescents did not consume milk and 197 (42.6%) citrus fruit at all. Many of the students 395(85.5%) used tea and coffee, and nearly two thirds 291 (63%) used immediately after taking other food. Majority of the participants, 338 (73.2%) and 364(78.8%) reported to have fruits and other vitamin A rich fruits and vegetables, respectively (Table 4).

**Table 4:** Dietary Diversity and pattern of the adolescents in DembiaDistrict, Northwest Ethiopia, 2017

Characteristics	Frequency(n)	Percent (%)
24 Hours meal frequency		
Two times	87	18.8
Three times	304	65.5
Four times	71	15.4
Feeding of meat for the last one weeks		
0–5 times	454	98.3
6–10 times	5	1.1
> 10 times	3	0.6
Drinking of tea and coffee		
No	67	14.5
yes	395	85.5
Timing of drinking of tea and coffee		
Before meal	104	22.5
After meal	291	63
Number of tea and coffee drinking per day		
1–6 cupper day	391	84.6
7–12 cupper day	4	0.9
Drinking of soft drinks		
No	272	58.9
yes	190	41.1
Number of soft drinks per day		
1–3 bottle per week	177	38.3
4–7 bottle per week	13	2.8
Citrus fruit		
One times per day	98	21.2
Two times per day	71	15.4
More than two times per day	11	2.4
One times per month	85	18.4
Never	197	42.6
Egg		
Always	8	1.7
One times per day	79	17.1
Two times per day	43	9.3
More than two times per day	33	7.1
One times per month	103	22.3
Never	196	42.2
Milk and milk product		
More than one times per day	19	4.1
One times per day	102	22.1
One times per week	130	28.1
Never	211	45.7
Dietary diversity score		
adequate dietary diversity	153	33.1
in adequate Dietary Diversity	309	66.9
Nutritional status		
well nourished	455	98.5
malnourished	7	1.5
House-hold Food security		
Food secure	397	85.9
food insecure	65	14.1

Almost all, 455(98.5%) of the adolescents were well nourished, and 397(85.9%) of the households were food secure (Table 4).

Concerning the families of adolescent, 279 (60.4%) used community pipe water. Two-thirds 312 (67.5%) of the parents were not using any treatment for water. Of the total 462 participants, 374 (81%) had toilets, while 209 (45.2%) had no hand washing practices after toilets (Table 5).

**Table 5:** Environmental sanitation and Hygiene of the adolescents in Dembia District, Northwest Ethiopia, 2017

Characteristics	Frequency(n)	Percent (%)
Source of water		
Protected spring water	19	4.1
River	9	1.9
Unprotected spring water	16	3.5
Pound water	19	4.1
Private pipe water	120	26
Community pipe water	279	60.4
Distance of water (round trip)		
≤ 30 min	338	73.2
> 30 min	124	26.8
Treatment of water		
Boiling	13	2.8
Water Agar	137	29.7
Never used	312	67.5
Availability of Toilet		
No	88	19
Yes	374	81
Field Defecation		
No	374	81
Yes	88	19
Practice of washing hand Before eating foods		
No	26	5.6
yes	436	94.4
After eating foods		
No	28	6.1
Yes	434	93.9
After using Toilet		
No	209	45.2
yes	253	54.8
Before Food processing		
No	210	45.5
yes	252	54.5
After Food processing		
No	55	11.9
Yes	407	88.1

### Prevalence of anemia

The overall prevalence of anemia was 25.5% (95% CI; 21.4, 29.2) in Dembia District. Out of the total anemic samples, 109(92.4%), 7(5.9%), and 2(1.7%) were mildly, moderately, and severely anemic, respectively.

### Factors associated with anemia

All the potential factors of anemia fulfilling the chi-square assumption were fitted into the bi-variable logistic regression model. Consequently, household food security, upper respiratory tract infection, living status of adolescence, dietary diversity score, and source of water were found with a *P*-value of< 0.2 in the bi-variable analysis and then fitted to the multivariable analysis. In the multivariable analysis, house hold food security, dietary diversity, and living with either of the two parents and guardians were significantly associated with anemia at a P-value of< 0.05.The odds of having anemia were 2.1 times ((AOR = 2.1;(95% CI; 1.3, 3.5)) higher among adolescents with inadequate dietary diversity compared to those with good dietary diversity. Likewise, the odds of having anemia were 2 times higher among adolescents who were living with either of the two parents compared to their counter parts ((AOR = 2.0;(95%CI; 1.14, 3.6)). The higher likelihood of anemia was demonstrated by the adolescents who were living with guardians as compared to those who lived with mothers and fathers (AOR = 2.4;(95% CI; 1.02, 5.6)). Finally, the odds of anemia increased among adolescents from food insecure households compared to their counterparts ((AOR = 1.9;(95% CI; 1.1, 3.5) (Table 6).

**Table 6:** Factors Associated with Anaemia among late Adolescent girls in Dembia District, Northwest Ethiopia, 2017

Variables	Categories	Anemia		COR	AOR
		Anemic	Non anemic		
Living status of the adolescence	With both family	84	285	1	1
	Either of two	23	45	1.7(1.0,3.03)	2(1.14,3.6)*
	Guardians	11	14	2.7(1.2,6.1)	2.4(1.02,5.6)*
Upper respiratory Tract	No	67	230	1	1
	yes	51	114	1.5(1.01,2.4)	1.4(0.9,2.3)
Dietary Diversity (score)	Adequate dietary diversity	27	126	1	1
	Inadequate dietary diversity	91	218	1.9(1.2,3.1)	2.1(1.3,3.5)*
Household food security	House hold food secure	93	304	1	1
	House hold food insecure	25	40	2.0(1.2,3.5)	1.9(1.1, 3.5)*
Source of water	Safe water	85	291	1	1
	Unsafe water	33	53	2.1(1.3,3.5)	1.2(0.6,2.1)

*p value less than 0.05

## Discussion

The prevalence of anemia is high in developing countries; it is estimated that 9 out of 10 anemia sufferers live in developing countries [29]. At the same time, about half of adolescent girls living in Sub-Saharan Africa are anemic [30].

The finding show that the overall prevalence of anemia among school adolescent girls was 25.5% (95% CI; 21.4, 29.2.) Based on WHO standards, this finding shows that anemia is a moderate public health concern among 15–19 years of age adolescent girls [9]. This finding is in line with that of Peri Urban Bangladesh(27%), Kenya (26.5%) [31] and the local report from Berhale District (22.8%) [19]. The possible reason might be low dietary intake of nutrient dense food groups, such as eggs, milk, and meat.

However, our result was found to be lower compared to those of studies conducted in Chennaie, India, which reported the overall prevalence of anemia to be 78.75%.The possible reason could be that a high proportion (13.3%) of the adolescents in Channie, India had massive menstrual bleeding disorder in the past two weeks prior to the data collection, while an insignificant (4.5%) number of participants experienced the problem in the current study. Such bleeding might explain a high prevalence of anemia in the latter study setting. On the other hand, the observed discrepancy could be related to the high magnitude of under nutrition (42.5%) in Channie, India compared to that of the current study (1.5%) [32],and the deficiency of micronutrient results malnutrition and contributes to the prevalence of anemia [33, 34] .

Similarly, compared to ours a higher prevalence of anemia (51.3%) was reported in Nepal. About 21% of the participants were found with worm infestation in the Nepal study, while it was 4.2% in the present study. Variations in hookworm infestation could explain the observed discrepancy in adolescent anemia [35]. This report was also lower than the previous local finding in Babile District (32%) [36].

However, the prevalence of anemia in this study was considerably lower than that of southern Iran (5.3%). The difference could be attributed to presence of iron folic acid supplementation; 46.3% of the Iranian adolescents were supplemented for iron and folic acid, while none of the participants were supplemented in the current study. Obviously, iron-folate supplementation boosts blood hemoglobin level which could be attributed to the lower prevalence of anemia in Iran [37].

Similarly, a lower prevalence of anemia (12%) was reported in Kebena, Addis Abeba. The low burden could be related to a better intake of micronutrient rich food in Kebena than adolescents of the present study. In Kebena, 65.0% of the adolescents ate meat and animal products at least once in a week, and 37.6% consumed vegetables more than 3 times per week [38].

The odds of having anemia were 2.1 times higher among adolescents with inadequate dietary diversity compared to those with good dietary diversity. This finding was supported by a study in Nigeria [39]. It was evident that diversification of diets enhances the micronutrient adequacy of the diet. Therefore, undiversified diet is a proxy indicator of poor micronutrient intake which increases the vulnerability of adolescents to anemia and other micronutrient deficiencies [40] . In another view this might be seasonality difference.

The odds of having anemia were 2 times higher among adolescents who were living with either of the two parents (father or mother) compared to their counterparts. Also, the odds of having anemia were 2.4 times higher among adolescents who lived with guardians compared to those who lived with mothers and fathers. The possible reason might be that out of the adolescents who lived with either of the two parents or guardians, 13.6 and 29.5% were from poor and Middle wealth tertile households, respectively. That is because economic status has a notable on the habit of eating balanced diet, especially the iron rich food groups.

The odds of having anemia were 1.9 times higher among adolescents from food insecure households compared to their counterparts. The finding was in agreement with what was reported in Bangladesh [41]. Previous researches demonstrated the positive effect of food security on dietary diversification and food intake. In fact, household food insecurity impairs micronutrient intake of household members which in turn increases the developing of anemia [42] .

## Conclusion

This study illustrated that; anemia is a moderate public health problem among adolescent girls in Dembia District. Household food security, dietary diversity, living with either of two patents and guardians only were significantly associated with anemia. Therefore, the government should focus on preventing food in security with increasing productivity to improve dietary diversification of the adolescent girls.

## Abbreviations

EDHS: Ethiopian demographic health survey
IDA: Iron Deficiency anemia
WHO: World Health Organization
